# Measurement of cyclosporin induced changes in P-glycoprotein function at the human blood-brain barrier using [^18^F]MC225 and PET

**DOI:** 10.1007/s00259-025-07320-0

**Published:** 2025-05-08

**Authors:** Pascalle Mossel, Giordana Salvi de Souza, Antoon T. M. Willemsen, Gilles N. Stormezand, Nicola A. Colabufo, Jun Toyohara, Hendrikus H. Boersma, Rudi A. J. O. Dierckx, Adriaan A. Lammertsma, Anna L. Bartels, Gert Luurtsema

**Affiliations:** 1 Department of Nuclear Medicine and Molecular Imaging, University of Groningen, University Medical Center Groningen, Groningen, The Netherlands; 2 Department of Radiology, Leiden University Medical Center, Leiden, The Netherlands; 3 School of Medicine, PUCRS, Porto Alegre, Brazil; 4 Dipartimento di Farmacia-Scienze del Farmaco, Università degli Studi di Bari “A. Moro”, via Orabona, 4, 70125 Bari, Italy; 5 Research Team for Neuroimaging, Tokyo Metropolitan Institute of Gerontology, 35-2 Sakae-cho, Itabashi-ku, Tokyo, 1730015 Japan; 6 Department of Neurology, Ommelander Ziekenhuis Groep, Scheemda, The Netherlands

**Keywords:** Positron emission tomography, Pharmacology, BBB, P-gp, CsA

## Abstract

**Introduction:**

P-glycoprotein (P-gp) or multidrug-resistance protein is one of the most extensively studied efflux transporters at the blood-brain barrier (BBB). Changes in P-gp function are associated with several neurodegenerative and psychiatric diseases, including Alzheimer’s disease, Parkinson’s disease and schizophrenia and with the bioavailability of several pharmaceuticals in the brain, causing multi-drug resistance or side effects. PET imaging can be used to measure the P-gp function *in vivo.* This study aims to validate [^18^F]MC225 as specific P-gp PET tracer with the use of cyclosporin as selective P-gp inhibitor.

**Methods:**

Fourteen healthy volunteers (age 67 ± 5y) were included. Subjects underwent twice a 60 min dynamic [^18^F]MC225 (200MBq) PET scan with continuous arterial blood sampling and a cerebral T1-weighted MRI as anatomical reference. During the second scan, in five subjects, cyclosporin was administered in a dose of 2.5 mg/kg/hour, starting 30 min prior to the scan, to inhibit the BBB P-gp function. Tissue time-activity curves of preselected brain regions (Hammer’s atlas) were fitted to a reversible two-tissue compartment model (2T4k) using the metabolite corrected plasma and uncorrected whole blood curves as input functions.

**Results:**

No significant difference was found in plasma kinetics, plasma curves, plasma-to-whole blood ratio, and the parent fraction of the baseline scans and scans after administration of cyclosporin. Volume of distribution values in whole brain grey matter showed a significant increase (6.18 ± 1.29 to 9.00 ± 1.29 mL·cm^− 3,^*p* = 0.03) after the administration of cyclosporin.

**Conclusion:**

The outcomes of the current study reflect the potential ability of [^18^F]MC225 to measure cyclosporin induced changes in P-gp function at the human BBB *in vivo.*

**Trial registration:**

EudraCT 2020-001564-28.

**Supplementary Information:**

The online version contains supplementary material available at 10.1007/s00259-025-07320-0.

## Introduction

The blood-brain barrier (BBB) plays a crucial role in maintaining homeostasis of the human brain by tightly regulating the passage of substances between the bloodstream and brain parenchyma [1]. P-glycoprotein (P-gp) or multidrug-resistance protein is one of the most extensively studied efflux transporters at the BBB. It is a member of the ATP-binding cassette (ABC) family and is highly expressed at the luminal side of the vessel walls of brain capillaries. It is responsible for the transport of a wide variety of neurotoxic substances, both endogenous and exogenous, and is therefore of great importance for protecting the brain [2]. Changes in P-gp function are associated with several neurodegenerative and psychiatric diseases, including Alzheimer’s disease, Parkinson’s disease and schizophrenia [3–8].

Permeability of the BBB is also one of the main factors influencing bioavailability of pharmaceuticals in the brain. A wide variety of structurally unrelated compounds are substrates of P-gp, and the penetration of many therapeutic agents into the CNS is significantly limited by P-gp, posing a significant challenge in the treatment of brain disorders such as brain tumours, epilepsy, and neurodegenerative diseases [9, 10].

The ability to quantify and visualise P-gp function at the BBB is important for understanding the role of this transporter in CNS physiology, pathology and drug-resistance. Positron emission tomography (PET) enables in vivo measurements of P-gp function and provides information on changes in transporter function. Clinically, PET imaging may be of value for studying the role of P-gp function in neurodegenerative diseases or as an in vivo screening tool for P-gp affinity of pharmaceuticals.

Several radiotracers have been developed and evaluated for PET imaging of P-gp. [13] These radioligands are typically derived from established P-gp substrates. To date, *(R)*-[^11^C]verapamil is the most widely used PET tracer for studying BBB P-gp function. In both preclinical and clinical studies, *(R)*-[^11^C]verapamil was used successfully to detect decreases in P-gp function after inhibition [11–14]. However, as *(R)*-[^11^C]verapamil is an avid P-gp substrate, tracer uptake at baseline levels is very low and therefore it is less suitable for measuring increases in P-gp function. [^18^F]MC225, a weak substrate, shows higher uptake at baseline levels, at least in preclinical studies, enabling measurements of both increases and decreases in P-gp function [15, 16].

In a previous human PET study regarding compartmental analysis and test-retest variability of [^18^F]MC225 measurements, a two-tissue compartment model (2T4k) model was selected as the best method for quantification of BBB P-gp function [17]. The use of this model provides several parameters that might be useful for quantification of P-gp function. The volume of distribution (V_T_) gives the brain-to-plasma partition coefficient, which is the total concentration of the PET tracer in tissue divided by the total concentration in plasma at equilibrium. For [^18^F]MC225, V_T_ seems to reflect P-gp function at the BBB best [17]. However, in preclinical studies and studies with *(R)*-[^11^C]verapamil also K_1_, reflecting the rate of influx, was studied as a parameter to assess P-gp function [18].

**Fig. 1:**
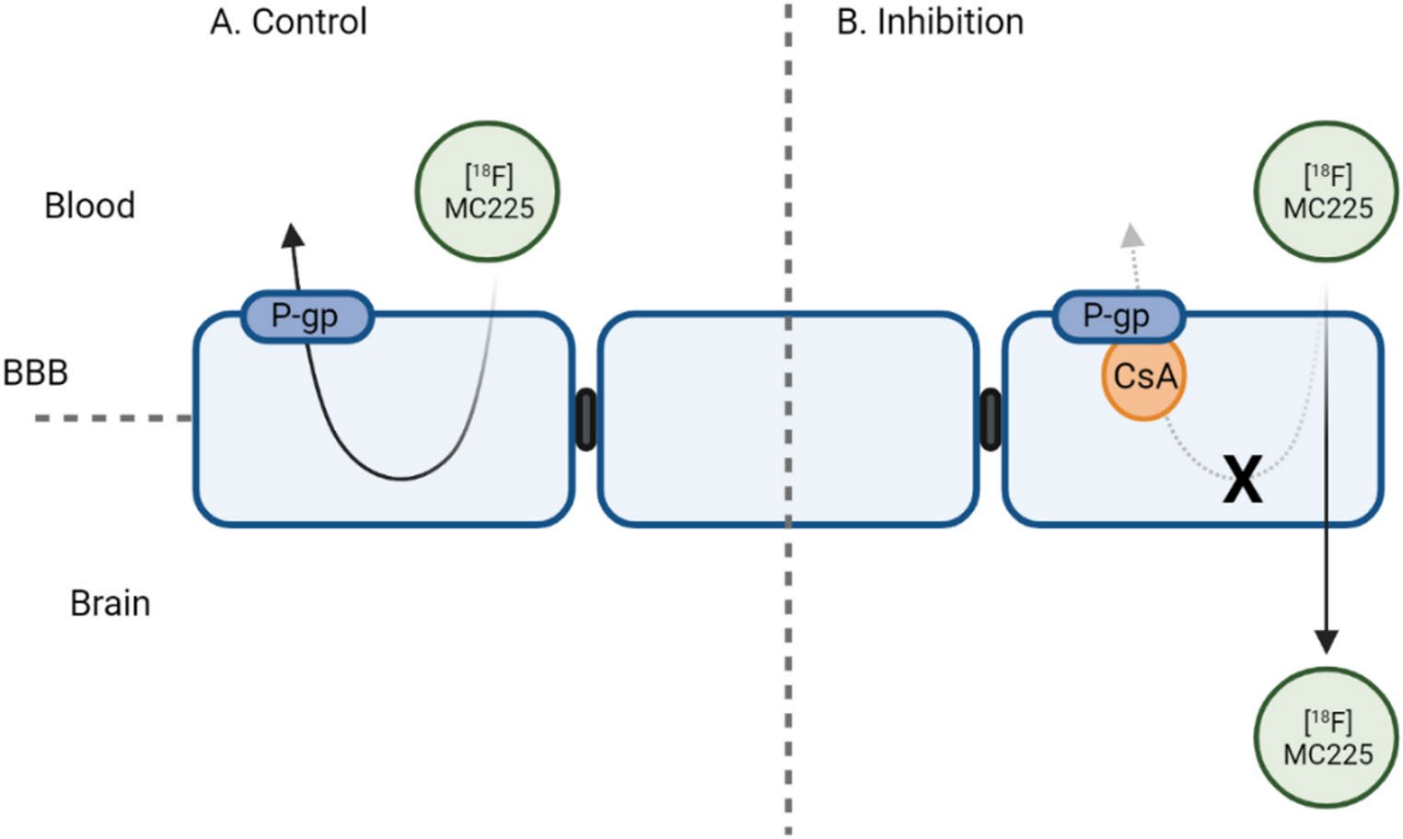
Schematic representation of P-gp inhibition with cyclosporin

The aim of the present study was to validate [^18^F]MC225 as specific P-gp PET tracer by measuring changes in P-gp function at the human BBB following administration of cyclosporin, a P-gp inhibitor (Fig. 1). The second aim was to assess whether a semi-quantitative approach without arterial sampling could be used for non-invasive clinical purposes. The final aim was to identify the optimal scanning procedure.

## Experimental design

### Study population

Fourteen healthy volunteers, 8 males and 6 females (mean age 67 ± 5 years), participated in the study. Nine of those volunteers only received a baseline scan, and five (3 male, 2 female, age 71 ± 4 years) were included in the cyclosporin protocol and underwent two [^18^F]MC225 PET scans (one at baseline and one after cyclosporin administration) with a minimum interval of two weeks between the two scans. Subjects were free of any medication affecting P-gp function and had no history of any neurological, psychiatric or psychological disease. One blood sample was obtained two weeks before the first scan to evaluate kidney function (eGFR). A Mini Mental State Examination (MMSE) score and a neurological examination were performed prior to the PET scans.

Written informed consent was obtained from all study participants. The study was approved by the Medical Ethics Review Committee of the University Medical Center Groningen, EudraCT 2020-001564-28, registered 25 May 2020.

### Radiochemistry

[^18^F]MC225 was manufactured at the Department of Nuclear Medicine and Molecular Imaging of the University Medical Center Groningen (EU-GMP production licence: 108964 F), as previously described [19]. For this human study, the tracer was validated in a GMP facility using an Eckert and Ziegler Pharmtracer Modular Synthesis Module. The molar activity was > 25,000 GBq/mmol and the radiochemical purity was > 98% in all cases.

### Imaging

Subjects underwent one or two 60 min [^18^F]MC225 (200 MBq) dynamic PET scans with continuous arterial blood sampling. [^18^F]MC225 was administered over 60 s via the antecubital vein. All PET scans were acquired on a Siemens Biograph Vision PET System (Siemens Healthcare GmbH, Erlangen, Germany). There was a minimum interval of two weeks between repeat [^18^F]MC225 scans to avoid any side-effects of the arterial sampling procedure.

A structural T1-weighted magnetic resonance imaging (MRI) scan was obtained prior to the first PET scan as an anatomical reference for the PET data. MRI scans were performed using a 3.0 T Siemens Magnetom Prisma (Siemens AG, Erlangen, Germany) with a 64-channel head coil and a voxel size of 1.5 × 1.5 × 3.0 mm.

### Arterial blood sampling

An arterial cannula was placed in the radial artery, and arterial blood was withdrawn continuously during the scan. A blood monitor (Twilite Sampling System, Swisstrace GmbH, Menzingen, Switzerland) was started 30 min prior to the scan, initially without blood running through the system to enable background correction. Then, thirty seconds before the start of the scan, the arterial cannula was connected to the blood monitor and on-line measurements started. In addition, five manual samples were taken at 5, 10, 20, 40 and 60 min after the start of the scan for calibration of the whole blood curve, and to correct for plasma-to-whole blood ratios and radiolabeled metabolites.

### Metabolite analysis

Parent fractions and radioactive metabolites of the manual plasma samples were analyzed for both baseline and cyclosporin scans. To precipitate plasma proteins, 50 µg of acetonitrile was added to the 50 µg plasma samples. After centrifuging the samples, metabolite analysis was performed using thin-layer chromatography (TLC), analyzing the supernatant of the samples with a mobile phase of methanol/ethyl acetate (10%/90%). Parent and metabolite fractions were then assessed using a phosphor imaging plate with a bioimaging analyzer (Amersham Typhoon, Global Life Sciences Solutions, USA).

### Cyclosporin

Cyclosporin was purchased from Sandimmun (50 mg concentrate for infusion, Novartis Pharma GmbH, Vienna, Austria). P-gp inhibition was reached by a continuous infusion of 2.5 mg/kg body weight/h, starting 30 min prior to the scan and with a maximum duration of 2 h [20]. 

## Data analysis

### Image reconstruction and co-registration

No preprocessing filter was applied to the [^18^F]MC225 scans. List mode from the emission scan were reconstructed (8 iterations, 5 subsets) into 26 frames (1 × 10, 10 × 5, 1 × 10, 2 × 30, 3 × 60, 2 × 150, 4 × 300, 3 × 600 s) with a voxel size of 0.9 × 0.9 × 0.9 mm. PET images were corrected for attenuation, randoms, dead-time, scatter, and decay. PET images were co-registered to the individual MRI images. Motion correction was performed as rigid transformation using the average of the first 12 frames as reference. After motion correction, images were spatially normalized to the Montreal Neurological Institute (MNI) space using PMOD (*version* 4.0, PMOD Technologies Ltd, Zürich, Switzerland). The co-registered MRI was segmented into grey matter, white matter and extra-cerebral fluid. The following brain regions of interest were defined based on the Hammer’s maximum probability atlas (Hammers N3083): amygdala, basal ganglia, brainstem, caudate nucleus, cerebellum, cingulate gyrus, corpus callosum, hippocampus, insula, occipital cortex, orbitofrontal cortex, pallidum, parietal cortex, putamen, temporal cortex, thalamus, whole brain white matter and whole brain grey matter. The pituitary gland was delineated using the method described by Ghazanfari et al. [21].Tissue time-activity curves (TACs) were derived in kBq/cc.

### Metabolite correction and plasma input function

The whole blood curve was converted to a plasma curve based on the exponential of the plasma-to-whole blood ratios of the manual samples. The metabolite fractions of the five manual samples were fitted to a Hill function. Then, the metabolite corrected plasma input curve was obtained by multiplying the total plasma curve with this Hill function. Both the metabolite corrected plasma curve and the whole blood TAC were used as input functions for pharmacokinetic (PK) modelling.

### Kinetic analysis

The PK analysis of the baseline scans has been described previously [22]. Similarly, tissue TAC’s of the cyclosporin scans were fitted to single tissue (1T2k) and both irreversible (2T3k) and reversible (2T4k) two-tissue compartment models using the metabolite corrected plasma and uncorrected whole blood curves as input functions. The fractional blood volume V_B_ was fixed to 5% for grey matter regions and to 3% for white matter regions or was estimated by including it as an additional fit parameter.

### SUV

SUV was calculated using the following formula

$$\:SUV=\frac{activity\:concentration\:(kBq/ml)}{injected\:activity\:\left(MBq\right)/\:body\:weight\:\left(kg\right)}$$

### Statistical analysis

The sample size was calculated using the G * Power 3.1R program [23]. This calculation was based on the experimental design and previous data on the use of [^18^F]MC225 in primates, considering both baseline and inhibition conditions [24]. An effect size of *d* = 8.875 (Cohen’s *d*) was estimated based on prior data. The power analysis was conducted with the following parameters: significance level α = 0.05, desired power (1 − β) = 0.80, non-centrality parameter δ = 8.875, and critical *t*-value = 4.30. The degrees of freedom (*df*) were 2, and the total sample size calculated was 4 (i.e., 2 individuals per condition). The resulting actual power was 0.97.

Descriptive data are described as mean ± standard deviation (SD) unless otherwise mentioned. Differences between baseline and cyclosporin scans were presented as percentage differences, calculated by dividing the absolute difference between the baseline and cyclosporin scans by the baseline value. A paired t-test was used to assess these differences. Correction for multiple comparisons was performed using the false discovery rate (FDR) approach [25]. The statistical threshold for significance was set at 0.05. Statistical analysis was performed using IBM SPSS Statistics version 23 (IBM Corp. 2015. IBM SPSS Statistics for Windows, Version 23.0. Armonk, NY). The Akaike Information Criteria (AIC) scores were used to identify the best model for quantifying P-gp function. AIC scores were converted to Akaike weights that can be interpreted as weight of evidence in favor of one of the models in the set [26].

Outcome parameters were defined according to the consensus nomenclature for in vivo imaging of reversibly binding radioligands [27].

## Results

### Safety analysis

Administration of [^18^F]MC225 was well tolerated by all subjects, and no complaints or symptoms could be attributed to tracer injection. After cyclosporin administration, two subjects reported hot flushes. Other side-effects associated with cyclosporin, such as weakness, dizziness, nausea, blurred vision and headache, remained absent in all subjects. No changes in heart rate and respiration rate were observed after administration of cyclosporin.

### Scan characteristics

No significant differences in administered activity (baseline 246 ± 72 MBq, cyclosporin 224 ± 101 MBq),molar activity or the calculated amount of administered cold MC225 were found between baseline and cyclosporin scans.

### Metabolite and free plasma fraction

**Fig. 2:**
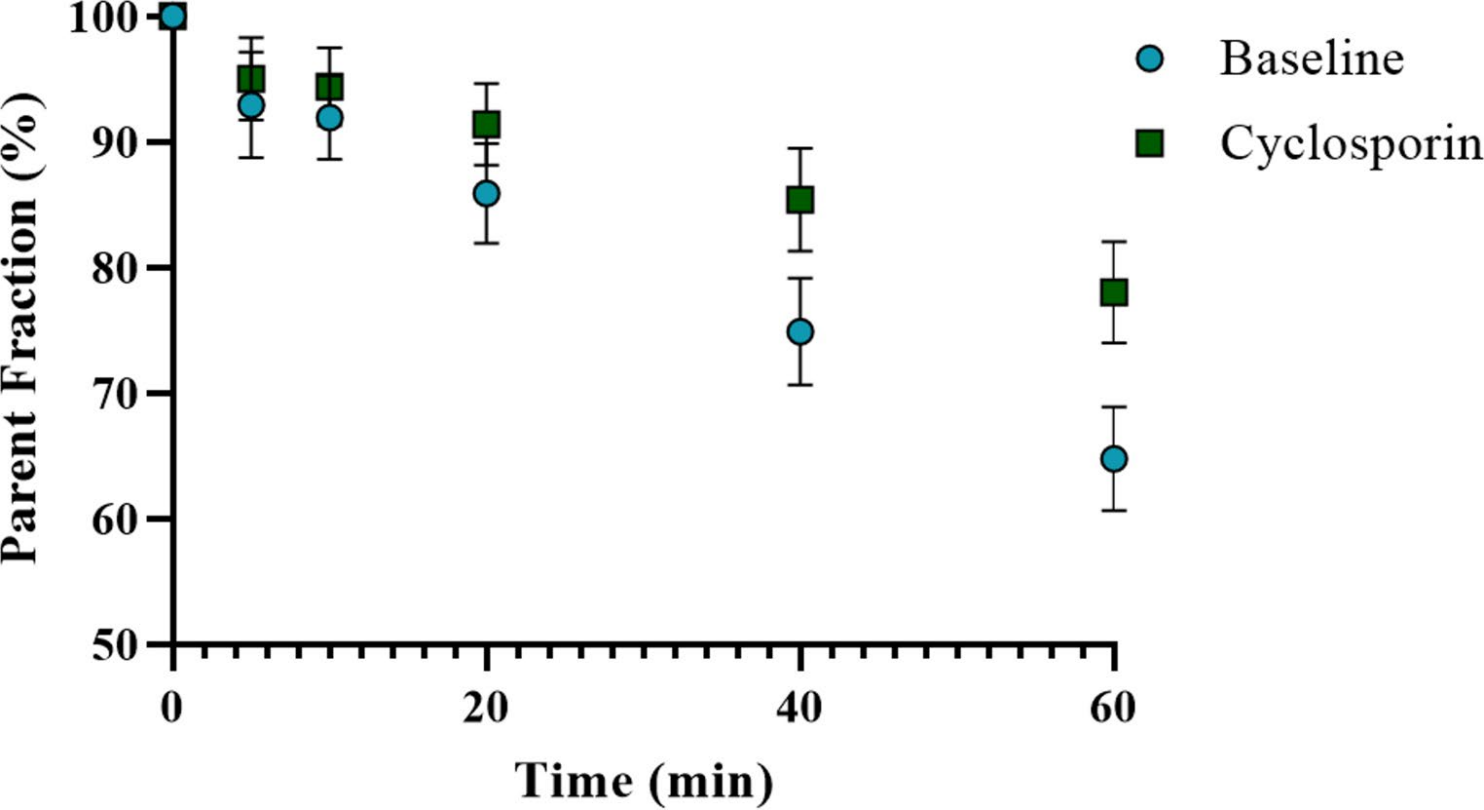
[^18^F]MC225 parent fraction in baseline and inhibition scans

**Fig. 3:**
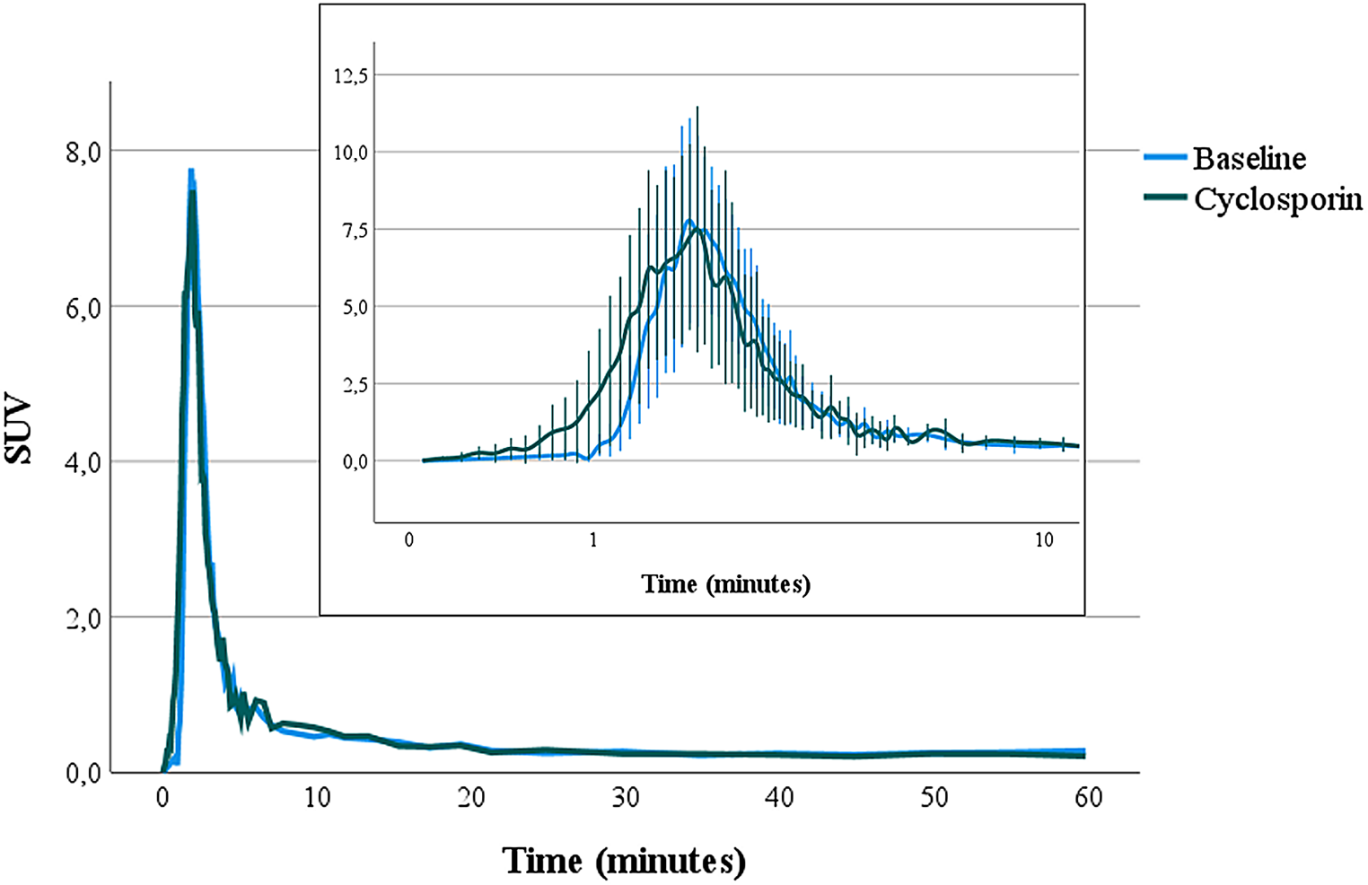
Mean ± SD of uncorrected plasma TAC, expressed in SUV (g/mL), of the baseline and cyclosporin scans

Figure 2 shows the metabolite fraction patterns of baseline and cyclosporin scans at different times during the scan. There was a significant difference in parent fraction at 60 min between baseline (68 ± 4%) and cyclosporin (78 ± 5%) scans (*p = 0.012*). No significant difference was found in the area under the curve (AUC) values of the uncorrected SUV plasma curves before (32.5 ± 5.3) g·mL^− 1^·min and after cyclosporin administration (31.4 ± 5.8) g·mL^− 1^·min (*p* = 0.99) (Fig. 3). Also, no significant difference was found in the AUC of the metabolite corrected plasma input function between the scans at baseline (79.3 ± 17.7) kBq·mL^− 1^·min and after cyclosporin administration (85.9 ± 53.3) kBq·mL^− 1^·min (*p* = 0.813).

The whole brain grey matter AUC was also evaluated, with values of 278.0 ± 47.9 kBq·mL^− 1^·min (94.9 ± 13.4 g·mL^− 1^·min) at baseline and 336.3 ± 209.6 kBq·mL^− 1^·min (111.5 ± 26.8 g·mL^− 1^·min) after cyclosporin administration (*p = 0.437* and *p* = 0.625). Supplementary data S2 presents the mean TACs for the whole brain grey matter region at baseline and cyclosporin scans. The AUC ratio between whole brain grey matter and the metabolite-corrected plasma input function was 3.51 ± 2.70 at baseline and 3.92 ± 3.94 after cyclosporin administration.

### Model selection and AIC

Baseline [^18^F]MC225 scans were analyzed using a 2T4k model, based on previous evidence that this model generated the best fits [22]. For reduced scan durations (10, 20 and 40 min), mean Akaike weights are shown in supplementary data S1. For the 60 min cyclosporin scans both AIC weights and visual inspection of the fits showed a preference for the 2T4k model.

For 20 and 10 min scan durations of both baseline and cyclosporin scans, the 2T4k model with fitted V_B_ also showed the highest AIC weights. For the 40 min scan duration the 2T4k with estimated V_B_ showed the best fit for the baseline scans, but the cyclosporin scans showed a preference for the 2T3K model with estimated V_B_. For the 60 min cyclosporin scans, the 2T4k with fixed V_B_parameter showed the best fits.

### Regional outcomes of cyclosporin Inhibition

**Fig. 4:**
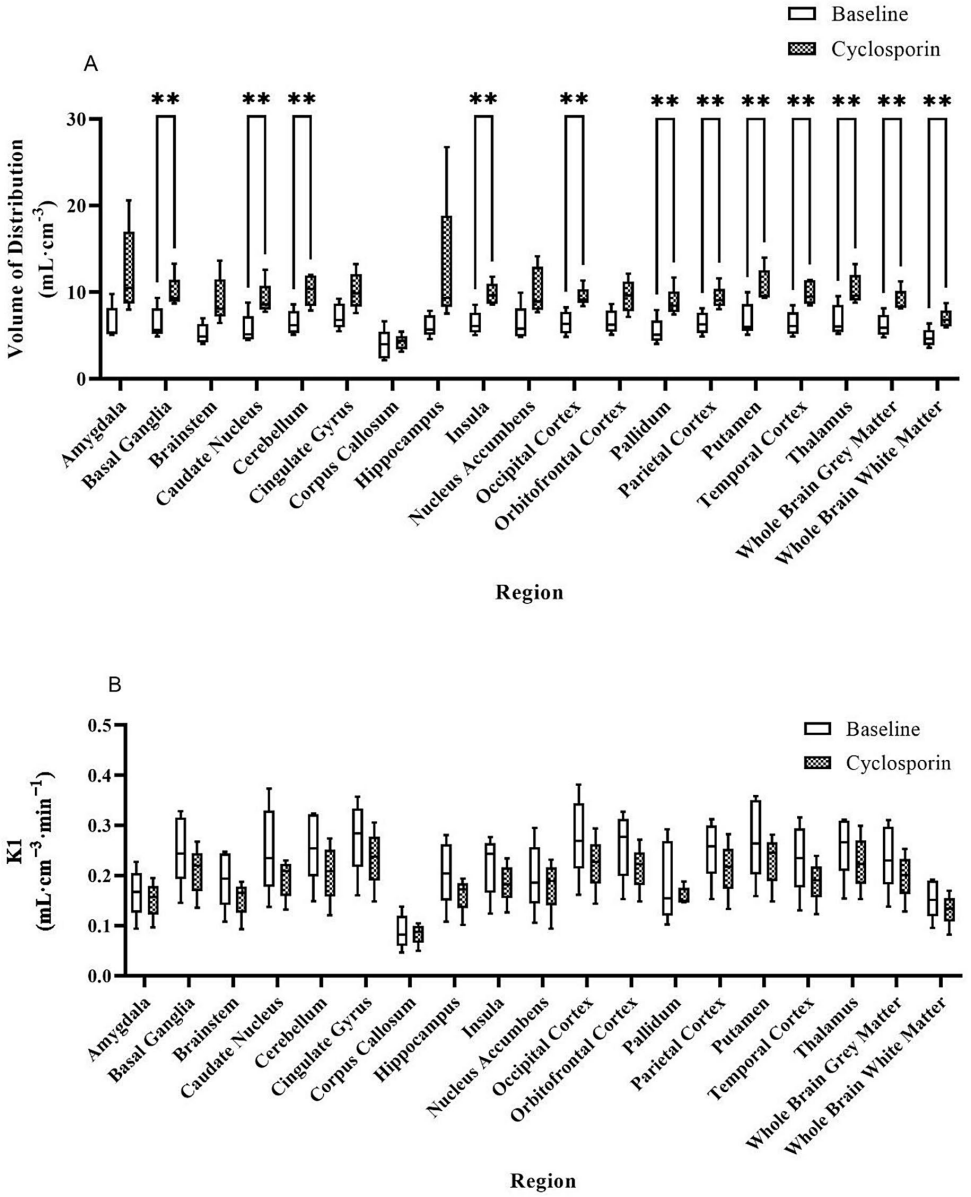
(**A**) V_T_ and (**B**) K_1_ outcome parameters before and after inhibition of the P-gp function with cyclosporin. **p < 0.001

Table 1; Fig. 4 show the (A) V_T_ and (B) K_1_ values of [^18^F]MC225 in preselected brain regions, obtained using a 2T4k model with estimated V_B_. At baseline, the highest V_T_ values were found in the cingulate gyrus (7.22 ± 1.33 mL·cm^− **3**^) (Table 1), and the lowest in the corpus callosum (3.92 ± 1.59 mL·cm^− **3**^). After cyclosporin administration, the highest V_T_ values were found in the hippocampus (12.72 ± 7.09 mL·cm^− **3**^) and the lowest in the corpus callosum (4.21 ± 0.77 mL·cm^− 3^). A significant difference was found between whole brain grey matter at baseline (6.18 ± 1.29 mL·cm^− **3**^) and after cyclosporin (9.00 ± 1.29 mL·cm^− **3**^), *p* = 0.003(Fig. 4). The most pronounced difference in V_T_ before (6.11 ± 1.13 mL·cm^− **3**^) and after (12.72 ± 7.09 mL·cm^− **3**^) cyclosporin administration was found in the hippocampus. However, due to the large SD for this region, this difference in uptake was not significant. The most pronounced significant V_T_ difference was found in the amygdala (6.39 ± 1.78 mL·cm^− **3**^ at baseline and 12.36 ± 4.47 mL·cm^− **3**^ after inhibition). For the whole brain grey matter region, the increase in V_T_ was 45 ± 22%.

For the K_1_ values, the K_1_ is slightly lower for every brain region, although no significant differences were present in the K_1_ values before and after administration of cyclosporin. Also, for the other micro parameters (k_2_, k_3_, and k_4_), no significant differences were found between baseline and cyclosporin scans (supplementary data S3 and S4). Supplementary data S5 shows the percentage standard error (%SE) of estimated parameters (V_T_, K_1_, k_2_, k_3_, and k_4_). Supplementary data S6 presents the changes in each subject’s outcome parameters (V_T_, K_1_, k_2_, k_3_, and k_4_) before and after inhibition.

**Table 1:** Regional outcome parameters before and after cyclosporine induced Inhibition of P-gp, obtained using a reversible two tissue compartment model (2T4k). Values in bold present *p* < 0.001

Region	V_T_ (mL·cm^− 3^)			K_1_/k_2_			k_3_/k_4_			SUV (g/mL)		
	Baseline	Cyclo-sporin	% Difference	Baseline	Cyclo-sporin	% Difference	Baseline	Cyclo-sporin	% Difference	Baseline	Cyclo- sporin	% Difference
*Amygdala*	6.39 ± 1.78	12.36 ± 4.47	93 ± 94	1.09 ± 0.40	1.08 ± 0.48	-13 ± 4338	5.36 ± 1.79	13.24 ± 7.08	133 ± 52	1.20 ± 0.20	1.55 ± 0.27	29 ± 13
*Basal Ganglia*	**6.46 ± 1.59**	**9.98 ± 1.68**	54 ± 22	1.47 ± 0.74	1.76 ± 0.68	7 ± 51	4.15 ± 1.42	5.85 ± 1.95	49 ± 54	1.38 ± 0.27	1.89 ± 0.38	37 ± 17
*Brainstem*	5.20 ± 1.06	9.09 ± 2.47	75 ± 54	1.02 ± 0.28	1.36 ± 0.59	10 ± 39	4.35 ± 0.86	7.40 ± 3.32	72 ± 81	1.09 ± 0.17	1.43 ± 0.28	31 ± 18
*Caudate Nucleus*	**5.76 ± 1.57**	**9.23 ± 1.73**	60 ± 19	1.23 ± 0.58	1.91 ± 0.66	32 ± 29	4.55 ± 1.87	4.66 ± 1.47	13 ± 34	1.26 ± 0.28	1.80 ± 0.39	43 ± 18
*Cerebellum*	**6.49 ± 1.25**	**10.21 ± 1.63**	57 ± 31	1.43 ± 0.49	2.24 ± 0.90	28 ± 27	3.89 ± 0.95	4.52 ± 1.64	18 ± 36	1.39 ± 0.22	1.86 ± 0.38	33 ± 22
*Cingulate Gyrus*	7.22 ± 1.33	10.15 ± 1.90	40 ± 32	1.47 ± 0.50	1.87 ± 0.67	13 ± 34	4.22 ± 0.90	5.28 ± 1.49	29 ± 37	1.50 ± 0.21	2.01 ± 0.47	33 ± 26
*Corpus Callosum*	3.92 ± 1.59	4.21 ± 0.77	7 ± 49	0.67 ± 0.23	0.57 ± 0.22	-27 ± 43	5.00 ± 1.80	7.93 ± 2.85	77 ± 72	0.61 ± 0.13	0.74 ± 0.20	21 ± 35
*Hippocampus*	6.11 ± 1.13	12.72 ± 7.09	108 ± 130	1.21 ± 0.39	1.46 ± 0.57	5 ± 37	4.40 ± 0.94	10.37 ± 7.25	136 ± 173	1.23 ±.17	1.55 ± 0.27	26 ± 16
*Insula*	**6.42 ± 1.21**	**9.78 ± 1.17**	52 ± 25	1.25 ± 0.44	1.62 ± 0.80	8 ± 33	4.54 ± 1.17	6.74 ± 2.64	47 ± 36	1.32 ± 0.18	1.76 ± 0.33	33 ± 19
*Nucleus Accumbens*	6.38 ± 1.87	9.94 ± 2.50	55 ± 23	1.32 ± 0.67	1.59 ± 0.088	-21 ± 78	4.67 ± 1.87	8.74 ± 4.38	85 ± 132	1.25 ± 0.26	1.72 ± 0.33	37 ± 14
*Occipital Cortex*	**6.47 ± 1.20**	**9.46 ± 1.00**	46 ± 21	1.56 ± 0.58	1.98 ± 0.72	15 ± 29	3.55 ± 0.97	4.70 ± 1.41	35 ± 28	1.40 ± 0.20	1.89 ± 0.35	36 ± 22
*Orbitofrontal Cortex*	**6.59 ± 1.22**	**9.54 ± 1.68**	44 ± 32	1.39 ± 0.51	1.99 ± 0.99	16 ± 37	4.22 ± 1.15	4.93 ± 1.81	25 ± 48	1.42 ± 0.21	1.90 ± 0.45	34 ± 26
*Pallidum*	**5.48 ± 1.34**	**8.80 ± 1.49**	60 ± 26	1.06 ± 0.39	1.23 ± 0.56	2 ± 31	4.77 ± 1.86	8.99 ± 5.49	83 ± 47	1.11 ± 0.25	1.47 ± 0.32	32 ± 23
*Parietal Cortex*	**6.44 ± 1.14**	**9.33 ± 1.21**	44 ± 23	1.46 ± 0.43	1.75 ± 0.54	10 ± 27	3.65 ± 0.77	5.14 ± 1.38	41 ± 24	1.39 ± 0.19	1.88 ± 0.40	35 ± 24
*Putamen*	**6.88 ± 1.71**	**10.70 ± ** **1.76**	55 ± 22	1.31 ± 0.45	1.73 ± 0.53	20 ± 28	4.65 ± 1.25	6.11 ± 1.91	31 ± 18	1.48 ± 0.31	2.03 ± 0.41	37 ± 17
*Temporal Cortex*	**6.37 ± 1.24**	**9.91 ± 1.24**	55 ± 26	1.23 ± 0.39	1.73 ± 0.63	22 ± 24	4.53 ± 01.04	5.95 ± 1.93	30 ± 23	1.33 ± 0.19	1.76 ± 0.30	33 ± 19
*Thalamus*	**6.79 ± 1.59**	**10.33 ± ** **1.50**	52 ± 27	1.56 ± 0.69	2.04 ± 0.95	5 ± 61	3.84 ± 0.83	5.62 ± 2.16	52 ± 66	1.43 ± 0.26	1.95 ± 0.37	36 ± 18
*Whole Brain Grey Matter*	**6.18 ± 1.29**	**9.00 ± 1.29**	49 ± 23	1.27 ± 0.38	1.72 ± 0.76	14 ± 35	4.16 ± 0.93	5.48 ± 1.81	33 ± 38	1.31 ± 0.20	1.77 ± 0.35	34 ± 23
*Whole Brain White Matter*	**4.75 ± 0.93**	**6.93 ± 0.99**	45 ± 26	1.00 ± 0.18	1.11 ± 0.37	4 ± 26	3.91 ± 0.86	6.37 ± 1.99	61 ± 25	1.00 ± 0.14	1.29 ± 0.40	29 ± 35
*Pituitary gland*	16.49 ± 5.24	16.80 ± 2.64	2 ± 50	7.32 ± 4.15	4.91 ± 2.38	33 ± 43	2.46 ± 3.11	3.14 ± 1.92	28 ± 38	3.13 ± 0.88	3.53 ± 0.53	13 ± 40

### SUV

**Fig. 5:**
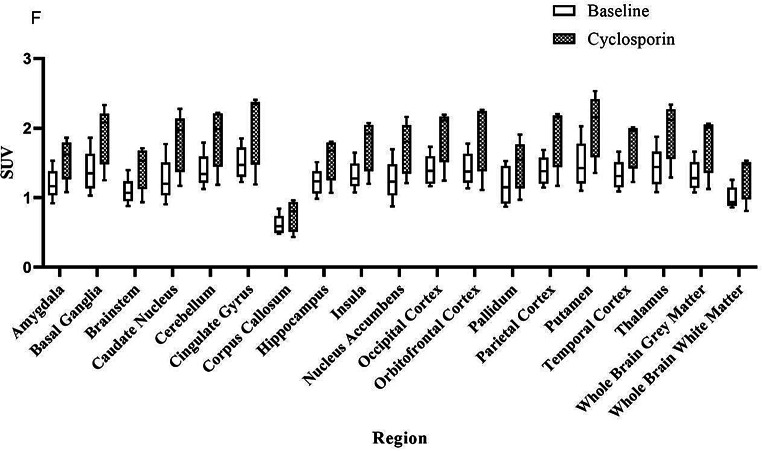
SUV (g/mL) outcomes at baseline levels and after administration of cyclosporin after 60 min of scan duration

**Fig. 6:**
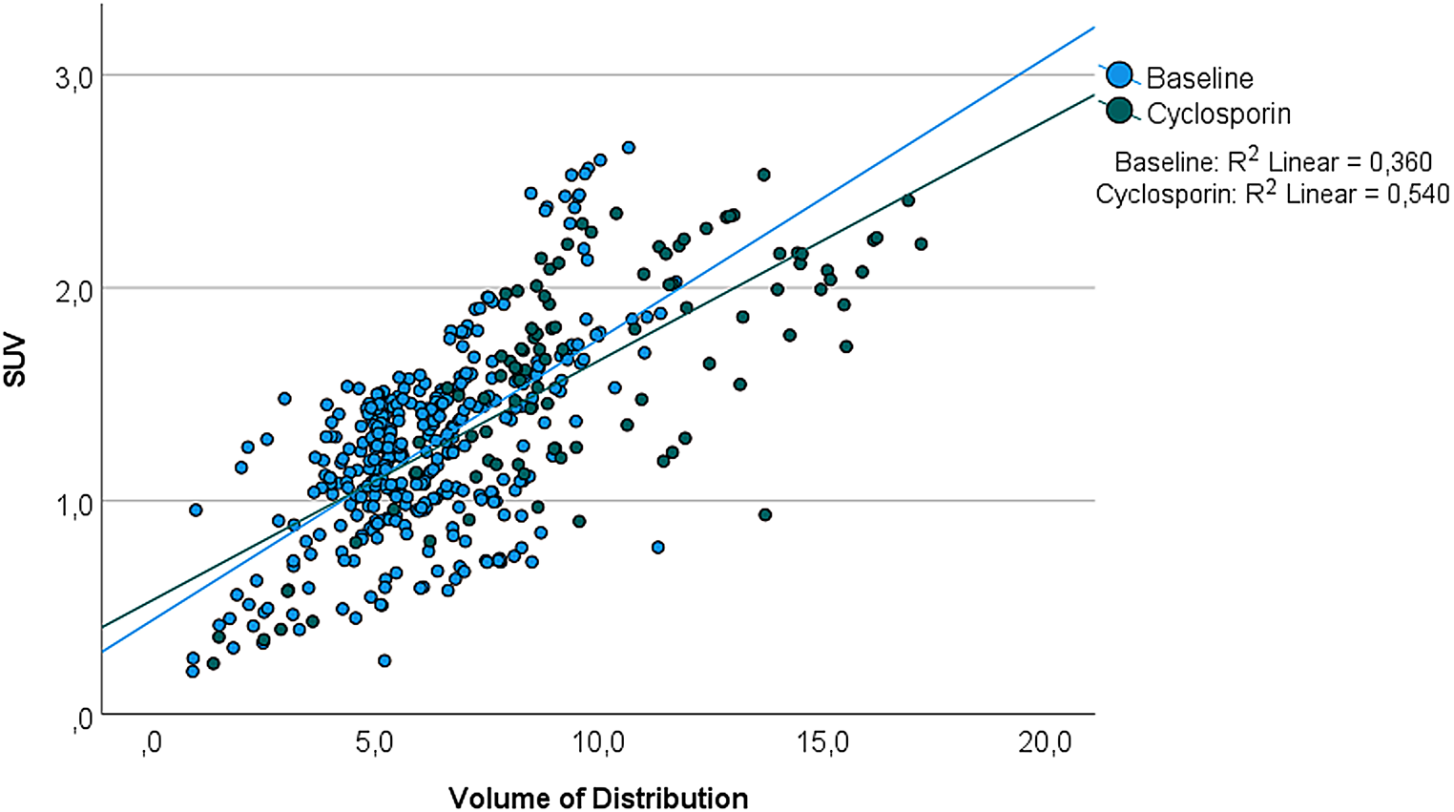
Correlation of the SUV and volume of distribution (V_T_) values of all brain regions for baseline and cyclosporin scans

Figure 5 shows SUV of [^18^F]MC225 in preselected brain regions. The highest SUV was found in cingulate gyrus (1.50 ± 0.21 g/mL) and putamen (1.48 ± 0.31 g/mL) and the lowest in corpus callosum (0.61 ± 0.13 g/mL). No significant difference was found between baseline and cyclosporin scans. The correlation between SUV and V_T_ was low for both baseline (R^2^ = 0.360) and cyclosporin scans (R^2^ = 0.540) (Fig. 6).

### Scan duration

As mentioned above, significant differences in V_T_ obtained with the 2T4k model were found after 60 min scan duration for the whole brain grey matter (baseline 6.18 ± 1.29 mL·cm^− 3^, cyclosporin 9.00 ± 1.29 mL·cm^**− 3**^, *p* = 0.03). V_T_ values obtained after 40 and 20 min of minutes of scan duration showed a strong correlation of R^2^ = 0.86 and R^2^ = 0.88, respectively with the values obtained at a 60 min scan duration. Ten minutes of scan duration showed a correlation of R^2^ = 0.33 (supplementary data S6). For all scan durations, the correlation to the 60 min scan was poorer for the cyclosporin scans.

**Fig. 7:**
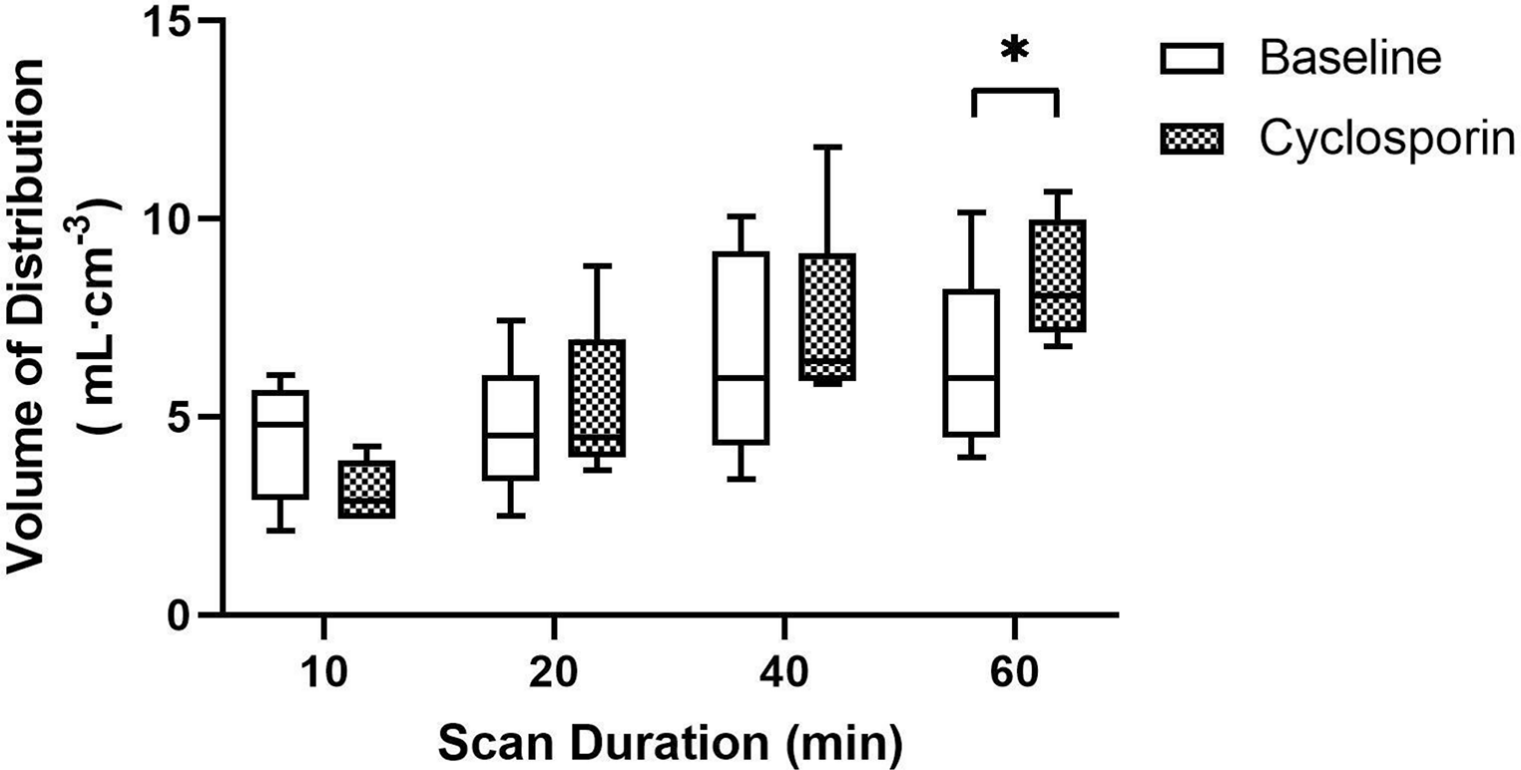
V_T_ values for the whole brain grey matter region after several scan durations

Figure 7 shows that shortening of the scan duration resulted in a non-significant difference for the 40 min (baseline 6.58 ± 2.63 mL·cm^− 3^, cyclosporin 7.30 ± 2.53 mL·cm^− 3^, *p* = 0.473), and 20 min (baseline 4.68 ± 1.77 mL·cm^**−** 3^, cyclosporin 5.28 ± 2.0 mL·cm^− 3^, *p* = 0.157) scans. After 10 min of scan duration the V_T_ value of the baseline scan was higher (4.39 ± 1.53 mL·cm^− 3^) than that of the cyclosporin scan (3.11 ± 0.78 mL·cm^− 3^, *p* = 0.053).

## Discussion

The most important finding of this study is the significant difference in V_T_ between baseline and cyclosporin scans for almost every brain region, including whole brain grey matter. This finding confirms that [^18^F]MC225 might be a suitable PET tracer to measure changes in P-gp function and, therefore, helpful in understanding molecular mechanisms of functional P-gp in the human brain. In a previous study, a test-retest variability of 28% in five healthy volunteers was observed [28]. The unidirectional increase in V_T_ after cyclosporin administration was 46% for whole brain grey matter, which was large enough to overcome this test-retest variability.

In a previous [^18^F]MC225 PK modeling study, a one-tissue compartment model resulted in poor fits compared with the 2T4kmodel, as indicated by higher AIC weights [28]. The present study shows that inhibition of P-gp function is also best described by the 2T4kmodel. Interestingly, after administration of cyclosporin, the preferred model switched from a 2T4k model with estimated V_B_ to a 2T4k model with fixed V_B_ for the 60 min scan duration. This might be explained by the fact that when the fixed V_B_ is more or less similar to the estimated V_B_, AIC will then assume the fixed V_B_ is lower, because of the penalty for extra estimated parameters in the model. The V_T_ was selected as a parameter of choice for both baseline and cyclosporin scans because of the higher precision compared with the micro parameters (K_1_, k_2_, k_3_, k_4_).

The preference for the 2T4k model reflects reversible uptake of the tracer in a second compartment behind the BBB. The biological correlation of this uptake is unknown and may not be directly related to P-gp. Despite the potential parallel process that may affect P-gp measurements at the BBB, a significant difference remains after cyclosporin administration. Future PK studies in this second compartment are needed to obtain a full understanding of the behavior of [^18^F]MC225 in the human brain.

The findings from the pituitary gland analysis further support the role of P-gp in modulating [^18^F]MC225 uptake. Since the pituitary gland is not protected by P-gp, the absence of significant V_T_ changes in this region strengthens the hypothesis that the observed increases in V_T_ in other brain regions are dependent on P-gp function.

In previous *(R)*-[^11^C]verapamil studies, both k_2_ and K_1_ have been reported as measures of BBB P-gp function [18, 29]. Muzi et al. found a K_1_ increase of 73% after modulating P-gp with cyclosporin when using [^11^C]verapamil, and Tournier et al. found a K_1_ increase of 9% with the use of [^11^C]metoclopramide [20, 30]. Studies evaluating P-gp function at the BBB in vitro suggest that P-gp transports its substrates from the BBB to the blood, even before they can enter the brain, which would be reflected in changes in K_1_ [31]. Surprisingly, in the current study, no differences in this micro parameter was found between baseline and cyclosporin scans, as significant changes were observed only in V_T_.

Another important consideration is the potential effect of cyclosporin on plasma protein binding, which could influence V_T_ measurements. Evidence from the literature suggests that cyclosporin can alter the plasma protein binding of PET tracers, leading to an increase in the plasma free fraction [32, 33]. A limitation of this study is that the free fraction of tracer in plasma was not determined and, therefore, an effect of cyclosporin on the free fraction cannot be excluded. Although a control experiment was performed (supplementary material S7), indicating that there was no substantial difference between control and cyclosporin treated plasma, further studies need to be performed to address this issue in more detail.

For clinical purposes, shorter scan durations (10, 20, and 40 min) would be of interest. Although differences between the baseline and cyclosporin scans were present for every scan duration, only 60 min scan duration showed a significant increase in V_T_. Since there is a continuous administration of cyclosporin during the scan, starting 30 min prior to the scan, an equilibrium is most likely already reached at the beginning of the scan. This implies that differences in V_T_ values for 40, 20, and 10 min scan durations are mainly caused by changes in the [^18^F]MC225 distribution and not by changes in the cyclosporin distribution.

One of the findings in the present study is a higher parent fraction in the cyclosporin compared with the baseline scans, which was significant at 60 min. This difference in metabolism is most likely caused by inhibition of cytochrome P450 following the cyclosporin administration [34]. CYP450 enzymes and P-gp are co-regulated via the pregnane xenobiotic receptor (PXR) and the constitutive androstane receptor (CAR) [35]. The higher parent fraction after cyclosporin inhibition does not affect the outcomes of the current study, as [^18^F]MC225 metabolites do not cross the BBB. However, SUV measurements will be affected by significant changes in metabolism.

Full quantification in PET imaging requires arterial blood sampling to estimate the plasma input curve. Unfortunately, arterial blood sampling is labor intensive and increases patient burden. For clinical use, non-invasive approaches for determining quantitative outcome parameters are essential. Full quantitative measurements without an arterial input function are possible if a suitable reference region is available. For [^18^F]MC225, however, such a reference region is not present in the brain, since changes in P-gp function are expected to affect influx and efflux of the tracer throughout the brain.

As an alternative, non-invasive semi-quantitative measurements were investigated. In the present study, however, only a low correlation was found between SUV and V_T_. The low agreement with the compartment modelling outcome values might be explained by various general sources of variability in SUV measurements. SUV is a simplified metric that gives information about the uptake of the radiotracer in the tissue of interest relative to the injected activity and does not provide information about complex physiological processes, including retention, clearance, metabolism and perfusion.

For less-invasive measurements of the P-gp function with the use of [^18^F]MC225 an image-derived input function might be an elegant non-invasive alternative, especially with the introduction of long axial field of view (LAFOV)PET scanners. These scanners include large blood pools, such as the aorta, in the same field of view as the target of interest, enabling accurate estimation of the whole-blood time-activity curve. In addition, venous blood samples could be a viable alternative to arterial samples for estimating the plasma parent fraction and plasma-to-whole blood ratio, offering a less invasive option for clinical settings.

*Concluding remarks and future perspectives -* Cyclosporin results in a significant increase in V_T_ values of [^18^F]MC225, reflecting the potential ability of [^18^F]MC225 to measure inhibition of P-gp function in vivo. The tracer holds promise for research, particularly in elucidating the role of P-gp at the BBB in neuropsychiatric diseases and in exploring new therapeutic approaches. While the clinical utility of [^18^F]MC225 in routine practice still requires further validation, it is a promising tool for investigating molecular mechanisms and advancing personalized medicine in neuropharmacology.

## Electronic supplementary material

Below is the link to the electronic supplementary material.

Supplementary Material 1

## Data Availability

The datasets generated during and/or analysed during the current study are available from the corresponding author on reasonable request.

## Abbreviations

1T2K: One-tissue Compartment Model
2T4K: Reversible two-tissue Compartment Model
2T3K: Irreversible two-tissue Compartment Model
ABC: ATP-ase Binding Cassette Transporter
AIC: Akaike Information Criteria
ATP: Adenosine Triphosphate
AUC: Area under the curve
BBB: Blood-brain barrier
CBF: Cerebral Blood Flow
FDR: False Discovery Rate
MRI: Magnetic Resonance Imaging
NHP: Non-Human Primate
P-gp: P-glycoprotein/Permeability Glycoprotein
PET: Positron Emission Tomography
PK: Pharmacokinetic
TAC: Time-activity curve
TLC: Thin Layer Chromotography
TRV: Test-Retest Variability
V_B_: Blood volume
