# Supplementary material for: Measurement of cyclosporin induced changes in P-glycoprotein function at the human blood-brain barrier using [18F]MC225 and PET
Source: Eur J Nucl Med Mol Imaging. 2025 May 8;52(12):4604–15. doi: 10.1007/s00259-025-07320-0 (PMC12491356; doi:10.1007/s00259-025-07320-0)
Supplement: Supplementary file 1 — Supplementary Material 1 [file 259_2025_7320_MOESM1_ESM.docx]

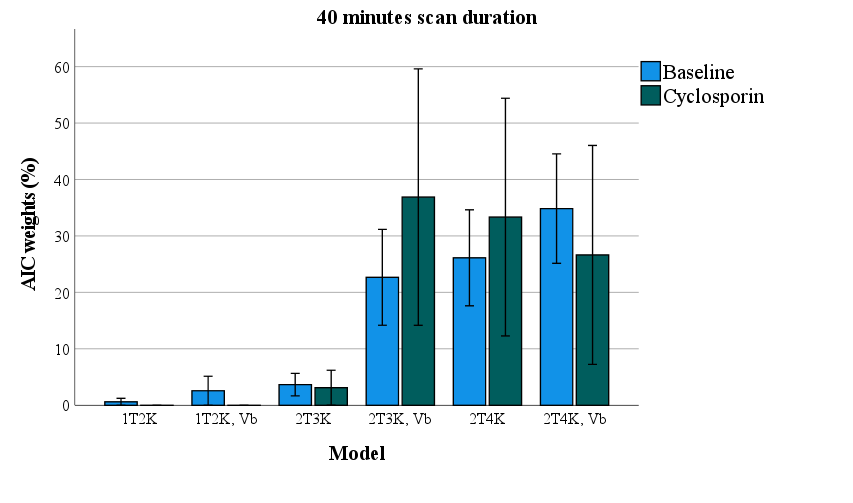
**
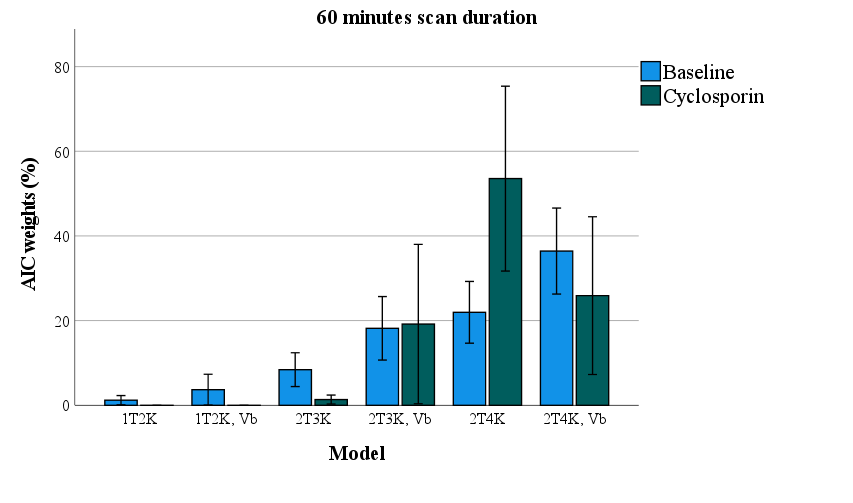
Supplementary data**


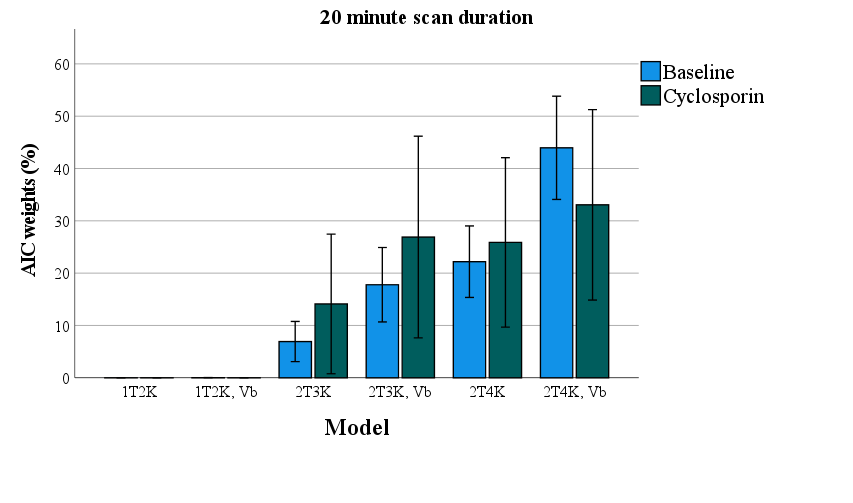


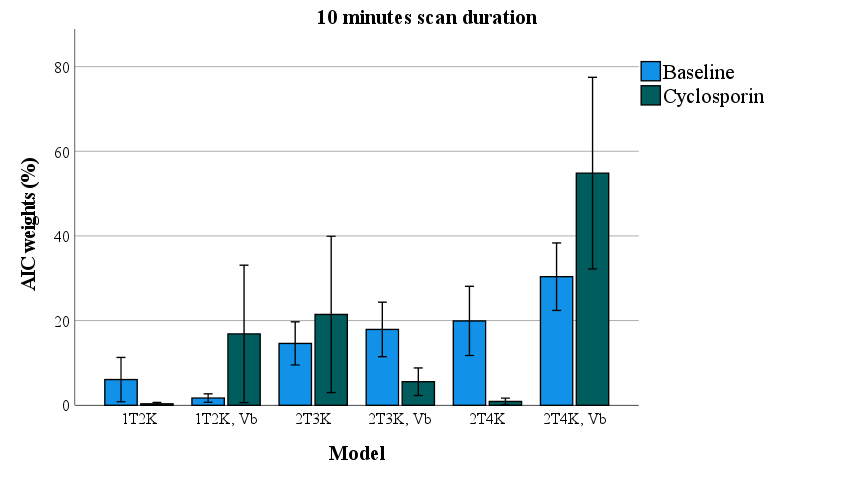


**Supplementary data S1** – AIC values for 60, 0, 20 and 10 minutes of scan duation

**Supplementary data S1** – AIC weighted values for 60, 40, 20, and 10 minutes of scan duration. 1T2K = 1 tissue compartment model, 2T3K – irreversible two-tissue compartment model, 2T4K = reversible two-tissue compartment model, Vb = fitted Vb parameter.

**Supplementary data S2** – Mean and SD of time activity curves (TAC) for the whole brain grey matter region in SUV (g/mL) for baseline and cyclosporin scans.

| **Brain regions** | **K_1_ (mL*cm^-3^*min^-1^)** | | | **k_2_ (min^-1^)** | | | **k_3_ (min^-1^)** | | | **k_4_ (min^-1^)** | | |
| --- | --- | --- | --- | --- | --- | --- | --- | --- | --- | --- | --- | --- |
|  | **Baseline** | **Cyclo- sporin** | **% Difference** | **Baseline** | **Cyclo- sporin** | **% Difference** | **Baseline** | **Cyclo-sporin** | **%Difference** | **Baseline** | **Cyclo-sporin** | **% Difference** |
| *Amygdala* | 0.16±0.04 | 0.15±0.03 | -8±38 | 0.16±0.09 | 0.16±0.05 | -0.7±73 | 0.19±0.08 | 0.18±0.06 | 7±48 | 0.04±0.01 | 0.02±0.01 | -52±21 |
| *Basal Ganglia* | 0.25±0.06 | 0.20±0.04 | -17±33 | 0.24±0.11 | 0.12±0.025 | -47±48 | 0.21±0.10 | 0.16±0.05 | -5±53 | 0.05±0.01 | 0.03±0.01 | -41±18 |
| *Brainstem* | 0.19±0.05 | 0.15±0.03 | -20±39 | 0.24±0.11 | 0.14±0.05 | -42±65 | 0.18±0.05 | 0.15±0.08 | -13±47 | 0.04±0.01 | 0.02±0.01 | -43±26 |
| *Caudate Nucleus* | 0.24±0.08 | 0.19±0.03 | -22±33 | 0.29±0.18 | 0.11±0.02 | -60±36 | 0.24±0.13 | 0.14±0.05 | -25±39 | 0.05±0.01 | 0.03±0.01 | -39±19 |
| *Cerebellum* | 0.25±0.06 | 0.20±0.05 | -20±36 | 0.23±0.10 | 0.11±0.05 | -50±67 | 0.20±0.07 | 0.13±0.04 | -25±45 | 0.05±0.01 | 0.03±0.01 | -37±28 |
| *Cingulate Gyrus* | 0.27±0.07 | 0.23±0.05 | -15±36 | 0.22±0.10 | 0.13±0.04 | -37±68 | 0.21±0.06 | 0.16±0.03 | -14±34 | 0.05±0.02 | 0.03±0.01 | -36±10 |
| *Corpus Callosum* | 0.08±0.03 | 0.08±0.02 | -5±50 | 0.15±0.08 | 0.18±0.07 | 16±91 | 0.11±0.04 | 0.14±0.04 | 46±40 | 0.03±0.01 | 0.02±0.01 | -9±29 |
| *Hippocampus* | 0.20±0.06 | 0.16±0.03 | -21±40 | 0.23±0.13 | 0.13±0.06 | -40±88 | 0.18±0.05 | 0.14±0.07 | -13±46 | 0.04±0.01 | 0.02±0.01 | -48±27 |
| *Insula* | 0.22±0.05 | 0.18±0.03 | -16±35 | 0.22±0.10 | 0.13±0.04 | -41±63 | 0.21±0.08 | 0.17±0.05 | -9±39 | 0.04±0.01 | 0.03±0.01 | -39±19 |
| *Nucleus Accumbens* | 0.19±0.06 | 0.18±0.05 | -8±48 | 0.19±0.14 | 0.16±0.08 | -16±127 | 0.19±0.11 | 0.16±0.08 | 40±99 | 0.04±0.01 | 0.01±0.01 | -46±30 |
| *Occipital Cortex* | 0.27±0.07 | 0.22±0.05 | -19±34 | 0.23±0.11 | 0.13±0.04 | -42±62 | 0.20±0.07 | 0.15±0.05 | -14±41 | 0.06±0.01 | 0.03±0.01 | -40±19 |
| *Orbitofrontal Cortex* | 0.26±0.06 | 0.21±0.04 | -17±32 | 0.24±0.11 | 0.13±0.02 | -46±50 | 0.22±0.09 | 0.14±0.05 | -17±44 | 0.05±0.01 | 0.03±0.01 | -39±13 |
| *Pallidum* | 0.18±0.07 | 0.15±0.02 | -15±35 | 0.27±0.24 | 0.20±0.11 | -27±53 | 0.18±0.08 | 0.16±0.04 | -1±38 | 0.03±0.01 | 0.02±0.01 | -42±24 |
| *Parietal Cortex* | 0.25±0.05 | 0.21±0.05 | -15±32 | 0.21±0.09 | 0.13±0.03 | -35±50 | 0.20±0.06 | 0.17±0.04 | -9±33 | 0.05±0.01 | 0.03±0.01 | -37±13 |
| *Putamen* | 0.27±0.07 | 0.23±0.04 | -15±34 | 0.24±0.11 | 0.14±0.03 | -40±50 | 0.23±0.08 | 0.17±0.06 | -18±35 | 0.05±0.01 | 0.02±0.01 | -39±20 |
| *Temporal Cortex* | 0.23±0.06 | 0.18±0.04 | -20±36 | 0.24±0.12 | 0.14±0.04 | -42±65 | 0.21±0.06 | 0.15±0.04 | -22±36 | 0.04±0.01 | 0.02±0.01 | -41±21 |
| *Thalamus* | 0.26±0.06 | 0.22±0.05 | -13±30 | 0.22±0.09 | 0.14±0.03 | -34±45 | 0.19±0.06 | 0.14±0.07 | -9±57 | 0.04±0.01 | 0.03±0.01 | -44±21 |
| *Whole Brain Grey Matter* | 0.23±0.06 | 0.19±0.04 | -16±34 | 0.22±0.10 | 0.13±0.03 | -3±60 | 0.21±0.07 | 0.15±0.06 | -13±45 | 0.05±0.01 | 0.03±0.01 | -40±17 |
| *Whole Brain White Matter* | 0.15±0.03 | 0.13±0.03 | -14±35 | 0.18±0.06 | 0.13±0.04 | -23±50 | 0.16±0.02 | 0.16±0.05 | 1±31 | 0.05±0.01 | 0.03±0.01 | -40±17 |
| *Pituitary gland* | 0.56±0.15 | 0.45±0.11 | 19±29 | 0.13±0.16 | 0.11±0.05 | 15±66 | 0.14±0.17 | 0.09±0.06 | 39±64 | 0.16±0.22 | 0.03±0.01 | 82±95 |

**Supplementary data S3** – Mean ± SD of regional microparameter outcomes for the baseline and cyclosporin scans.


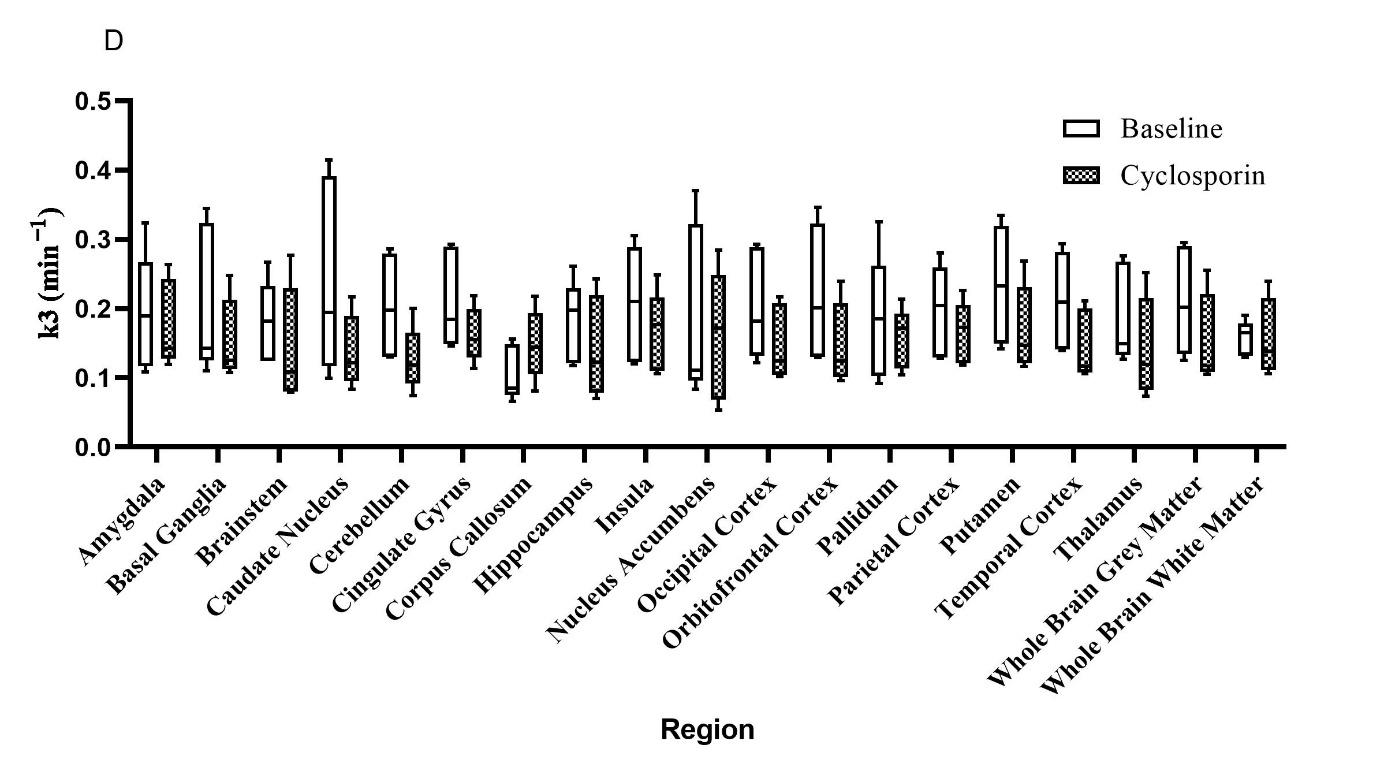


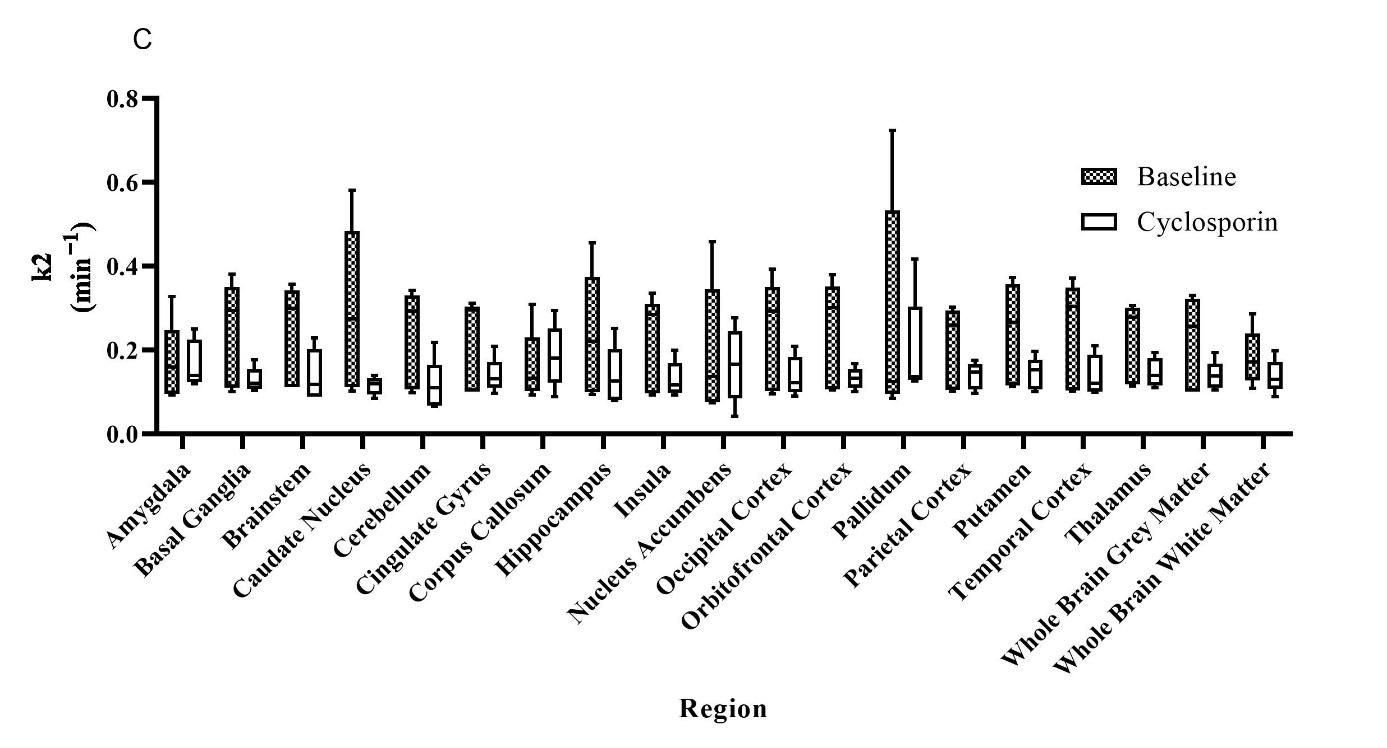


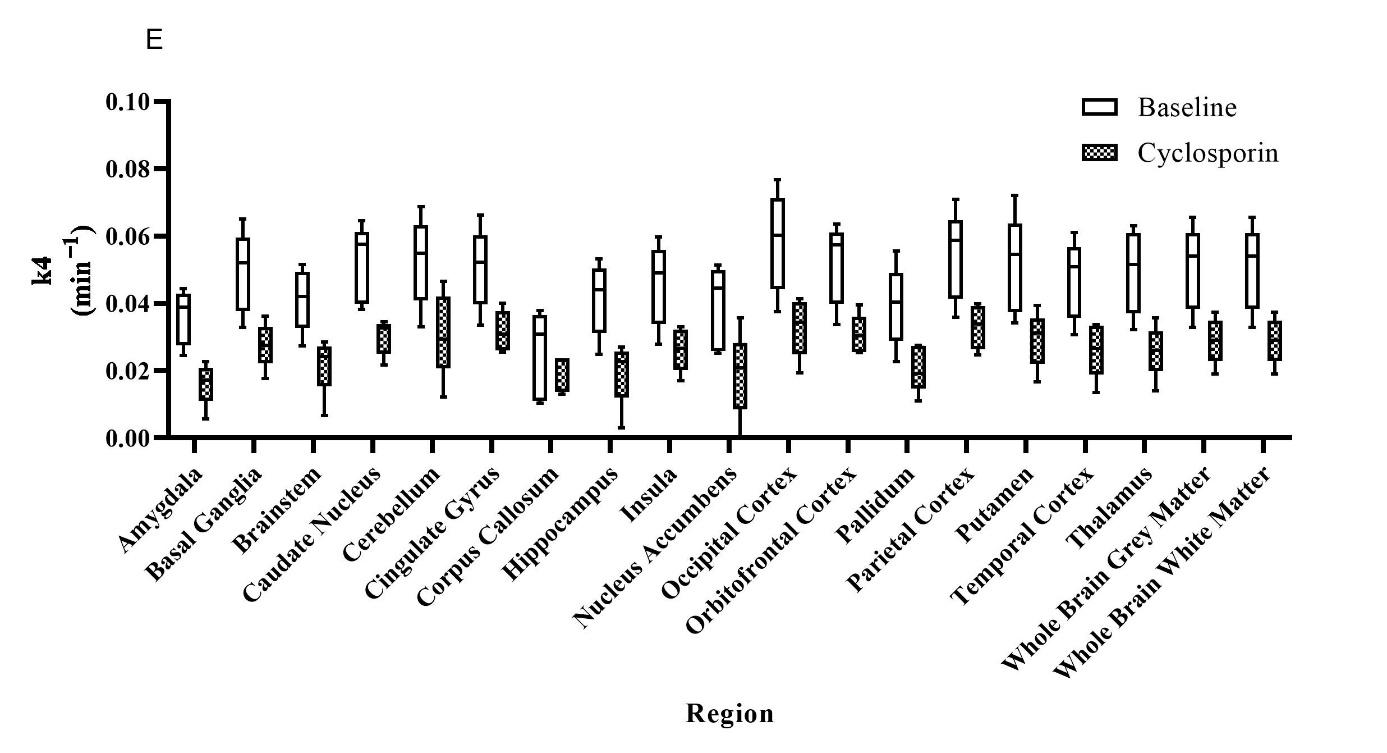


**Supplementary data S4** – k_2_, k_3_, and k_4_ values at baseline and cyclosporin scans.

| **Brain regions** | **V_T_ (mL*cm^-3^)** | | **K_1_ (mL*cm^-3^*min^-1^)** | | **k_2_ (min^-1^)** | | **k_3_ (min^-1^)** | | **k_4_ (min^-1^)** | |
| --- | --- | --- | --- | --- | --- | --- | --- | --- | --- | --- |
|  | **Baseline** | **Cyclosporin** | **Baseline** | **Cyclosporin** | **Baseline** | **Cyclosporin** | **Baseline** | **Cyclosporin** | **Baseline** | **Cyclosporin** |
| *Amygdala* | 4.7 ± 2.0 | 10.6 ± 9.6 | 5.6 ± 3.5 | 5.1 ± 1.5 | 21.0 ± 7.0 | 19.2 ± 8.9 | 16.3 ± 4.7 | 13.3 ± 4.6 | 11.2 ± 4.4 | 16.4 ± 11.5 |
| *Basal Ganglia* | 2.8 ± 1.7 | 3.2 ± 1.2 | 3.4 ± 2.0 | 2.5 ± 0.4 | 14.5 ± 7.7 | 9.2 ± 3.0 | 12.5 ± 5.6 | 7.6 ± 3.3 | 7.0 ± 3.1 | 7.5 ± 2.8 |
| *Brainstem* | 3.0 ± 1.6 | 7.3 ± 8.8 | 4.2 ± 2.5 | 3.2 ± 0.8 | 15.3 ± 6.8 | 11.9 ± 3.7 | 12.7 ± 5.1 | 10.2 ± 4.0 | 7.5 ± 3.3 | 12.2 ± 10.2 |
| *Caudate Nucleus* | 2.8 ± 1.3 | 4.7 ± 2.9 | 6.4 ± 4.6 | 2.8 ± 0.9 | 27.4 ± 13.2 | 13.8 ± 7.0 | 21.5 ± 8.2 | 14.5 ± 5.6 | 11.5 ± 6.3 | 10.6 ± 4.2 |
| *Cerebellum* | 2.7 ± 1.8 | 5.2 ± 5.0 | 3.9 ± 2.5 | 2.4 ± 0.4 | 18.1 ± 10.3 | 9.9 ± 4.8 | 16.7 ± 12.9 | 12.0 ± 4.6 | 8.0 ± 4.2 | 11.5 ± 7.0 |
| *Cingulate Gyrus* | 3.3 ± 2.0 | 2.6 ± 0.9 | 4.4 ± 2.7 | 3.1 ± 1.1 | 17.9 ± 9.9 | 12.7 ± 4.3 | 14.4 ± 6.0 | 10.7 ± 3.1 | 8.5 ± 3.7 | 6.8 ± 2.0 |
| *Corpus Callosum* | 10.1 ± 11.1 | 6.3 ± 6.0 | 4.6 ± 2.1 | 4.5 ± 1.1 | 21.5 ± 13.5 | 15.1 ± 6.8 | 20.7 ± 10.6 | 12.8 ± 6.8 | 20.3 ± 14.0 | 11.0 ± 9.1 |
| *Hippocampus* | 3.9 ± 2.3 | 16.8 ± 28.4 | 4.7 ± 2.9 | 3.8 ± 0.9 | 18.9 ± 8.1 | 14.8 ± 4.3 | 15.5 ± 5.4 | 12.8 ± 4.5 | 9.2 ± 4.1 | 23.3 ± 30.2 |
| *Insula* | 3.2 ± 2.5 | 3.5 ± 1.3 | 3.3 ± 1.9 | 3.7 ± 1.1 | 13.2 ± 5.0 | 14.3 ± 2.1 | 11.3 ± 4.5 | 11.3 ± 3.7 | 8.4 ± 3.9 | 8.4 ± 2.3 |
| *Nucleus Accumbens* | 16.0 ± 28.5 | 187219.6 ± 418619.0 | 7.3 ± 5.3 | 7.6 ± 2.1 | 31.4 ± 11.0 | 32.6 ± 14.4 | 34.2 ± 16.1 | 31.0 ± 20.9 | 34.6 ± 50.0 | 187238.7 ± 418643.6 |
| *Occipital Cortex* | 3.2 ± 2.5 | 3.0 ± 1.7 | 4.8 ± 4.4 | 2.9 ± 1.2 | 19.4 ± 15.9 | 10.9 ± 4.5 | 15.4 ± 7.5 | 9.6 ± 5.0 | 9.1 ± 4.7 | 8.3 ± 2.9 |
| *Orbitofrontal Cortex* | 2.9 ± 1.7 | 3.0 ± 1.4 | 3.7 ± 2.4 | 3.0 ± 0.4 | 15.8 ± 9.8 | 13.2 ± 6.5 | 15.1 ± 12.1 | 12.8 ± 6.2 | 7.3 ± 3.2 | 8.0 ± 3.1 |
| *Pallidum* | 4.2 ± 1.9 | 4.6 ± 2.7 | 4.4 ± 3.0 | 3.4 ± 1.1 | 17.8 ± 10.6 | 9.5 ± 5.5 | 18.6 ± 13.7 | 7.7 ± 5.5 | 10.4 ± 5.0 | 9.1 ± 4.2 |
| *Parietal Cortex* | 3.0 ± 2.0 | 3.3 ± 2.0 | 3.5 ± 2.2 | 3.0 ± 0.9 | 14.8 ± 7.7 | 12.7 ± 6.4 | 14.0 ± 7.2 | 10.7 ± 3.7 | 7.9 ± 4.2 | 7.6 ± 2.2 |
| *Putamen* | 3.0 ± 1.7 | 3.2 ± 1.8 | 4.4 ± 2.3 | 2.7 ± 0.8 | 22.1 ± 10.0 | 9.7 ± 4.1 | 18.8 ± 11.3 | 8.2 ± 4.7 | 7.8 ± 3.5 | 7.8 ± 2.4 |
| *Temporal Cortex* | 11.6 ± 12.7 | 24.7 ± 27.2 | 6.3 ± 3.1 | 6.5 ± 2.7 | 26.2 ± 7.1 | 20.4 ± 11.1 | 28.9 ± 12.1 | 18.6 ± 13.0 | 25.0 ± 21.4 | 33.1 ± 32.4 |
| *Thalamus* | 2.9 ± 1.7 | 4.2 ± 3.1 | 4.0 ± 2.1 | 3.1 ± 0.8 | 19.4 ± 9.0 | 11.6 ± 3.6 | 16.5 ± 7.6 | 9.9 ± 4.8 | 7.8 ± 3.0 | 9.3 ± 4.1 |
| *Whole Brain Grey Matter* | 3.1 ± 2.0 | 3.2 ± 1.5 | 3.9 ± 2.4 | 3.1 ± 0.7 | 15.8 ± 7.7 | 12.7 ± 3.6 | 13.9 ± 7.5 | 10.6 ± 3.8 | 7.9 ± 2.7 | 7.8 ± 2.3 |
| *Whole Brain White Matter* | 4.0 ± 3.4 | 4.8 ± 2.3 | 7.0 ± 4.9 | 7.0 ± 2.8 | 20.6 ± 8.0 | 20.8 ± 9.0 | 17.3 ± 11.0 | 14.5 ± 6.3 | 14.6 ± 12.3 | 10.2 ± 3.3 |
| *Pituitary gland* | 8.9 ± 5.2 | 23.0±23.6 | 8.6 ± 7.0 | 5.9 ± 3.1 | 86.3±97.9 | 23.7±14.1 | 708341.0±2124118 | 64.9±86.9 | 829.9±2405.0 | 79.2±123.5 |

**Supplementary data S5** – Mean ± SD of percentage standard error (%SE) of V_T_ and microparameter outcomes for the baseline and cyclosporin scans.


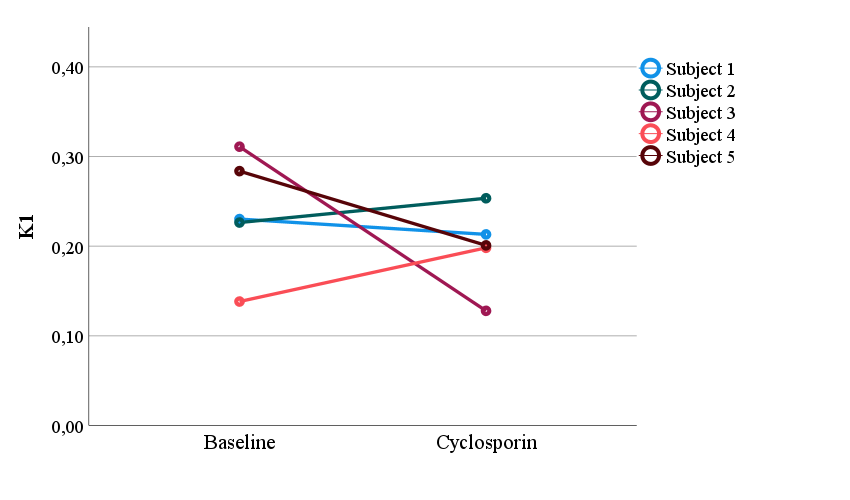

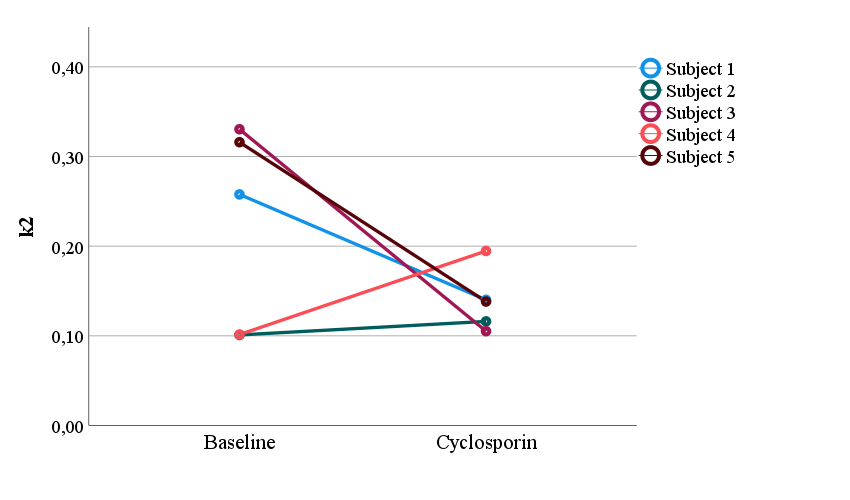

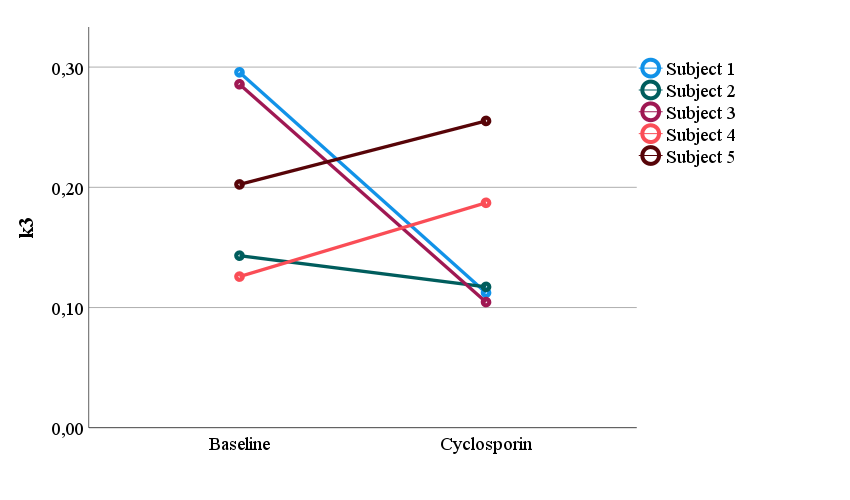

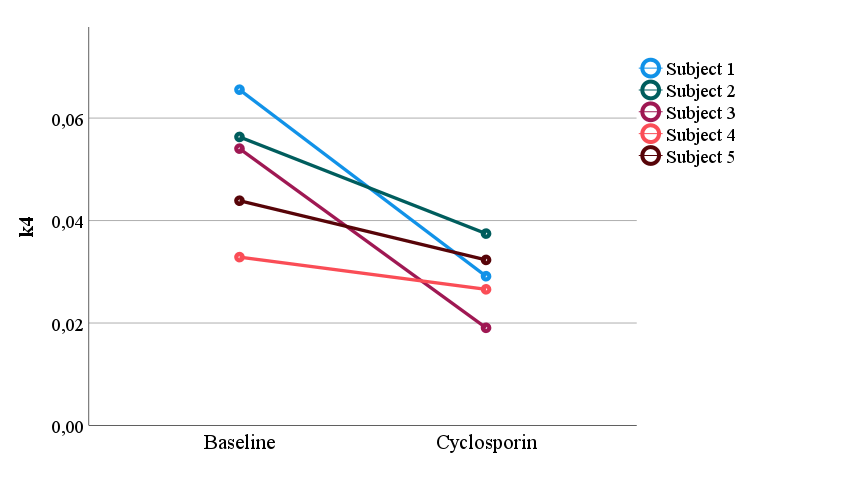

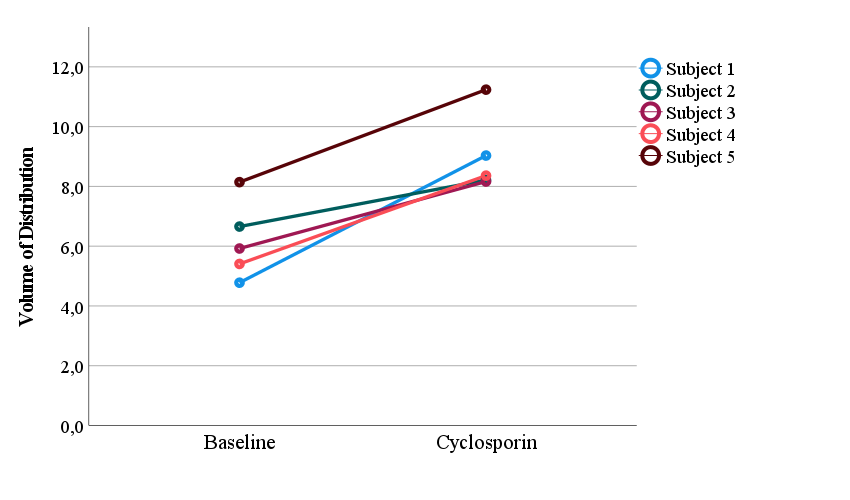


**Supplementary data S5** – Plots of individual subjects before and after cyclosporin administration


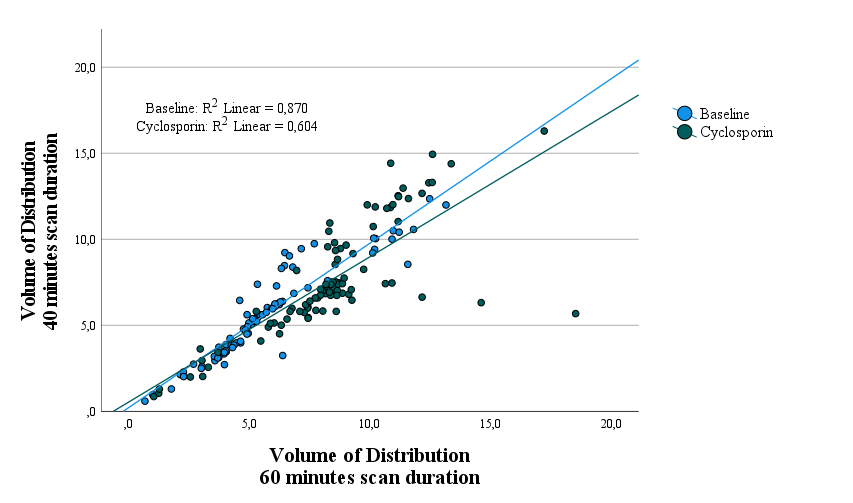


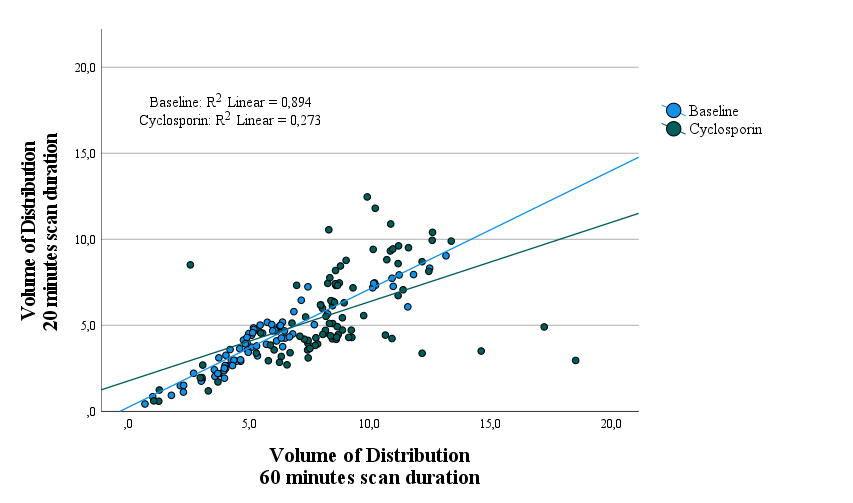


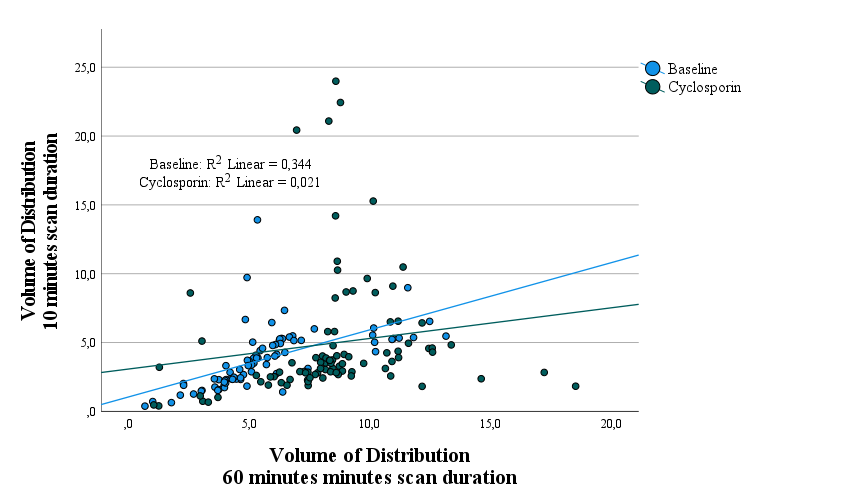


*Supplementary data S3 – Scatter plots of V_T_ 40, 20, and 10 minutes of scan duration and the standard of 60 minutes scan duration*

**Supplementary data S6** – Correlation plots for 40, 20, and 10 minutes of scan duration outcome parameters (SUV and V_T_).

**Title:**

Effect of cyclosporine on plasma binding of [^18^F]MC225.

**Materials and equipment:**

Cyclosporine A: Sandimmune for i.v. 50 mg/L

Gamma counter: Perkin Elmer (Revvity) 2480 gammacounter

Centrifuge: Mikro 20 Hettich centrifuge for eppendorfs

**Methodology**

Human plasma (450 μL) was treated with either 50 μL of Cyclosporine A (4 µmol/L, treatment group) or 50 μL of saline (control group) and incubated at 37 °C for 30 minutes (n=3, from 1 batch). Next, [^18^F]MC225 (100 kBq) was added to 500 μL of plasma and incubated at 37 °C for another 60 minutes. Plasma proteins were precipitated by adding 500 μL of acetonitrile, vortexing, and centrifuging at 4000 rpm for 10 minutes to separate the protein free supernatant from the plasma protein sediment. Radioactivity in both fractions was measured using a γ-counter. The ratio was calculated with the following formula:

$$=(\frac{Radioactivity supernatant}{weight of supernatant})/(\frac{Radioactivity sediment}{weight of sediment})$$

**Results/ Conclusion**

The protein free supernatant-to-sediment radioactivity ratio was 1.753 ± 0.173 for the cyclosporin treated group and 1.632 ± 0.050 for the control group. This indicates that there are no substantial differences between the two groups.

**Supplementary data S7** – Additional experiment cyclosporin effect on plasma binding of [^18^F]MC225
